# A Biomechanical Model of the Inner Ear: Numerical Simulation of the Caloric Test

**DOI:** 10.1155/2013/160205

**Published:** 2013-10-02

**Authors:** Shuang Shen, Yingxi Liu, Xiuzhen Sun, Wei Zhao, Yingfeng Su, Shen Yu, Wenlong Liu

**Affiliations:** ^1^State Key Laboratory of Structural Analysis for Industrial Equipment, Dalian University of Technology, Dalian 116024, China; ^2^Department of Otorhinolaryngology, The Second Hospital of Dalian Medical University, Dalian 116027, China; ^3^School of Information and Communication Engineering, Dalian University of Technology, Dalian 116024, China

## Abstract

Whether two vertical semicircular canals can receive thermal stimuli remains controversial. This study examined the caloric response in the three semicircular canals to the clinical hot caloric test using the finite element method. The results of the developed model showed the horizontal canal (HC) cupula maximally deflected to the utricle side by approximately 3 **μ**m during the hot supine test. The anterior canal cupula began to receive the caloric stimuli about 20 s after the HC cupula, and it maximally deflected to the canal side by 0.55 **μ**m. The posterior canal cupula did not receive caloric stimuli until approximately 40 s after the HC cupula, and it maximally deflected to the canal side by 0.34 **μ**m. Although the endolymph flow and the cupular deformation change with respect to the head position during the test, the supine test ensures the maximal caloric response in the HC, but no substantial improvement for the responses of the two vertical canals was observed. In conclusion, while the usual supine test is the optimum test for evaluating the functions of the inner ear, more irrigation time is needed in order to effectively clinically examine the vertical canals.

## 1. Introduction 

The caloric stimulation test has been widely used to examine vestibular performance in clinical medicine. This procedure uses thermal irrigation of the ear canal with cold and/or hot fluid (water or air) to elicit a vestibular signal. The intensity of the signal can be measured from the associated eye movement or nystagmus. The caloric test was reported to be the only examination that can stimulate individual vestibular systems [1]. Although the caloric test has important clinical implications, it only investigates the low frequency response mainly of the horizontal semicircular canals [2, 3]. 

In recent years, many researchers have focused on whether the caloric test can evaluate the function of vertical semicircular canals. Yagi et al. [4] reported that caloric nystagmus contains vertical and torsional components and posited that their origins are the posterior and superior semicircular canals. Fetter et al. [5] evaluated the functions of vertical canals by taking three-dimensional recordings during caloric stimulation for different head positions. Furthermore, Aw et al. [6] determined that, compared to the lateral canal's contribution to the overall caloric response magnitude during the reorientation from a horizontal to vertical position, the anterior canal (AC) contributed about one-third and the posterior canal (PC) about one-tenth the contribution from the lateral canal. In addition, Aoki et al. [7] also discovered that vertical canals functioned during the caloric test with a head tilt. However, neither the posterior nor superior semicircular canal was observed to receive a caloric effect by Ichijo [1] and Ichijo [8]. These studies indicate that the vertical and torsional components originate in the horizontal canal (HC). According to Aoki et al. [9], among 200 ears, 50% exhibited no vertical component of the caloric response, 45% exhibited a small upbeating component, and 5% exhibited a small downbeating component. 

Unfortunately, the physical origins of the caloric test have also been an ongoing controversy. Based on experimental and theoretical analyses, Gentine et al. [10–13] and later both Zucca et al. [14] and Valli et al. [15] concluded that the gravity-dependent buoyancy force is the largest contributor to the caloric response. However, Kassemi et al. [16] argued that the previous researchers did not incorporate the effects of fluid dynamics, and they further discovered through a finite element (FE) study that the dominant mechanism is the natural convection driven by the temperature-dependent variation in the bulk fluid. Kassemi et al. [16] also established that the effect of the expansion convection is approximately two orders of magnitude smaller than that of the natural convection. Despite its inability to fully explain the effects of orientation on the caloric nystagmus, Barany's convection hypothesis remains largely acceptable to the majority of the vestibular community [17]. Nevertheless, the caloric response in vertical canals still has an insufficient quantitative description. 

This study aimed to develop an FE model for a human membranous labyrinth, including the utricle and three membranous semicircular canals, and to examine the vestibular response to a caloric test with different head positions. The mechanisms of endolymph convection due to the buoyancy force, endolymph expansion, and cupular expansion are considered simultaneously for their contribution to the caloric response of semicircular canals.

## 2. Materials and Methods 

**Figure 1:**
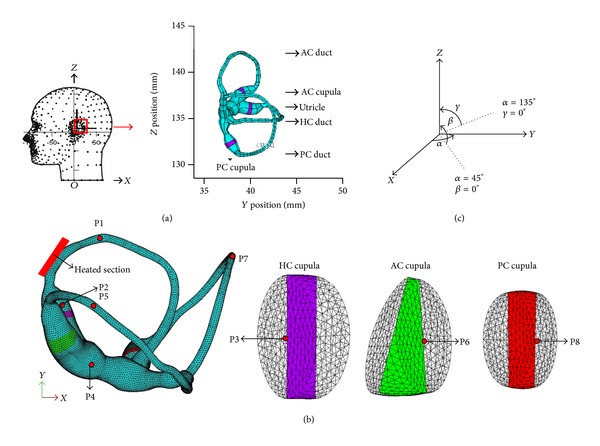
(a) The reconstructed model of a healthy human membranous labyrinth with dimension extracted from the report by Ifediba et al. [18]. The reconstruction is presented with +*X* as posterior, +*Y* as right lateral, and +*Z* as superior. (b) Finite element mesh used for the simulations. (c) The gravitational direction during the caloric test. The gravity is parallel to the +*X*-axis when the head is in the supine position.

We reconstructed a healthy human right membranous labyrinth based on the morphologically descriptive model provided by [18]. The computational model consists of the utricular cavity, the HC, the AC, the PC, and their ampullae; see Figure 1. Perpendicular to the longitudinal axis of the canal, three cupulae hypothetically traversed their respective ampulla. The utriculo-endolymphatic valve (UEV) opening is assumed to be located in the anteroinferior wall of the utricle, and the utricle is assumed to be separate from the saccule [19–23]. 

In a caloric test, the ear canal is irrigated with fluid (water or air) that is 7°C above the nominal body temperature of 37°C. The segment of the HC that is closest to the bone (see Figure 1) experiences a temperature rise of approximately 0.5–1°C, according to Cawthorne and Cobb [24]. Thus, the endolymph flow, driven by the natural convection due to gravity and expansive convection, which in turn is due to the thermal expansion of the endolymph, can be described by the conservation equations of mass, momentum, and energy. In this study, an Arbitrary Lagrangian Eulerian (ALE) approach is adopted, and the endolymph is assumed to be a slightly compressible Newtonian fluid with constant properties, except for the density, which is a linear function of the temperature but not pressure. All the governing equations are listed below. Consider.

(1) $$\begin{array}{c} \frac{\partial \rho }{\partial t}+\nabla \cdot \left(\rho u\right)=0, \end{array}$$

(2) $$\begin{array}{c} \rho_{o}\frac{\partial u}{\partial t}+\rho_{o}\left(u-u_{m}\right)\nabla \cdot u=-\nabla p+\mu \nabla^{2}u+\rho g, \end{array}$$

(3) $$\begin{array}{c} \rho_{o}c\frac{\partial T}{\partial t}+\rho_{o}c\left(u-u_{m}\right)\nabla \cdot T=k\nabla^{2}T, \end{array}$$

(4) $$\begin{array}{c} \rho =\rho_{o}\left[1-\beta_{T}\left(T-T_{o}\right)\right]. \end{array}$$

Here, *ρ*, *u*, *p*, and *T* are the density, velocity, pressure, and temperature, respectively. Time is denoted by *t*, and *μ*, *β*_*T*_, *c*, and *k* are the dynamic viscosity, thermal expansion coefficient, heat capacity, and thermal conductivity of the fluid, respectively. The gravitational acceleration is represented by *g*, and the subscripts *o* and *m* refer to the reference and mesh values, respectively. The values for the endolymph conductivity, volume expansion coefficient, reference density, and viscosity are taken from measured data provided by Steer et al. [25], and the specific heat is assumed to be equal to the value for water [26].

Nonslip boundary conditions are applied at all the physical boundaries of the canal, except for the wetted surfaces of the cupula, which are specified through the coupling with the structural equations and at the outlet of the UEV. The nodal velocities at the UEV opening are allowed to be unconstrained. The inner ear is initially assumed to be at body temperature, namely, 37°C, which is also the reference temperature, *T*_*o*_. At time zero, a 1°C temperature rise is imposed on the section of the HC, as indicated in Figure 1. This thermal impulse is maintained throughout the transient simulation.

The heat transfer through the cupulae is governed by an energy conservation equation similar to (3). Stimulated by the thermal expansion, thermal strain, *ε*_*T*_, is generated on the cupulae, which is incorporated into the initial condition. The thermal strain can be written as follows:

(5) $$\begin{array}{c} \varepsilon_{T}=\alpha_{T}\left(T-T_{o}\right). \end{array}$$

Here, *α*_*T*_ is the thermal expansion of the cupula. The conductivity, density, specific heat, and thermal expansion of the cupula are assumed to be the same as the values for the endolymph. Concurrently, driven by the endolymph flow and the thermal expansion, the motion of the three cupulae can be written in terms of the displacement vector, *d*, as

(6) $$\begin{array}{c} \rho_{c}\frac{\partial^{2}d}{\partial t^{2}}=\nabla \cdot \sigma , \end{array}$$

where *ρ*_*c*_ is the cupula density and *σ* is the Cauchy stress. In this study, the cupula is assumed to be a linearly elastic material undergoing a large deformation that involves small strains. The movement of the cupula is restricted at the peripheral boundaries, where a tight seal is formed with the ampulla wall. The cupula displacements at these boundaries are set to zero. For the wetted surfaces of the endolymph-cupula boundaries, the strong coupling between the fluid flow and the structural deformations is rigorously preserved via a balance of the traction forces and continuity of the velocities and the displacements. All the structural properties of the cupula used in the present analysis are extracted from [16, 26] and are listed in Table 1.

The FE model was further implemented using ADINA software (v8.7, ADINA R&D Inc.). Comprehensive grid convergence tests were performed to ensure sufficient spatial resolution of the generated solutions, and the results presented herein were obtained with a grid involving 7496 tetrahedral solid elements with 2078 nodes for the 3 cupulae and 68277 tetrahedral fluid elements with 18106 nodes for the endolymph. 

Finally, the fluid and solid equations were solved in parallel using a step-by-step iterative solution algorithm. The time step is set to 1 s, and the total time of the simulation is 300 s. At each time step, the convergence tolerance was 0.001 for the velocity and displacement norms and 0.01 for the fluid-solid surface norm. The endolymph flow and the cupular deformation responses to a set of caloric tests with different head positions are then examined. Different head positions are evaluated in order to maximize the caloric response of an individual canal while simultaneously minimizing the response of the other two canals. The gravitational direction is assumed to be parallel to the anatomical canal plane as much as possible (plane *α* for the HC, plane *β* for the PC, and plane *γ* for the AC), as shown in Figure 1. 

## 3. Results 

**Figure 2:**
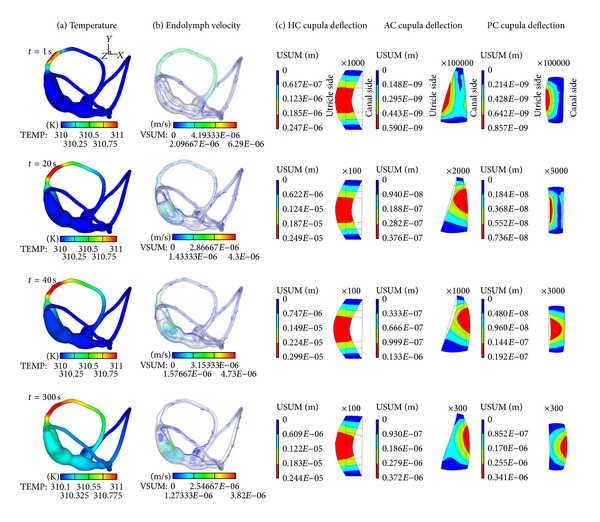
Time sequences of the temperature field (a), the endolymph streamline (b), and cupula displacement fields (c) during a hot supine caloric test with Δ*T* = 1°C.

**Figure 3:**
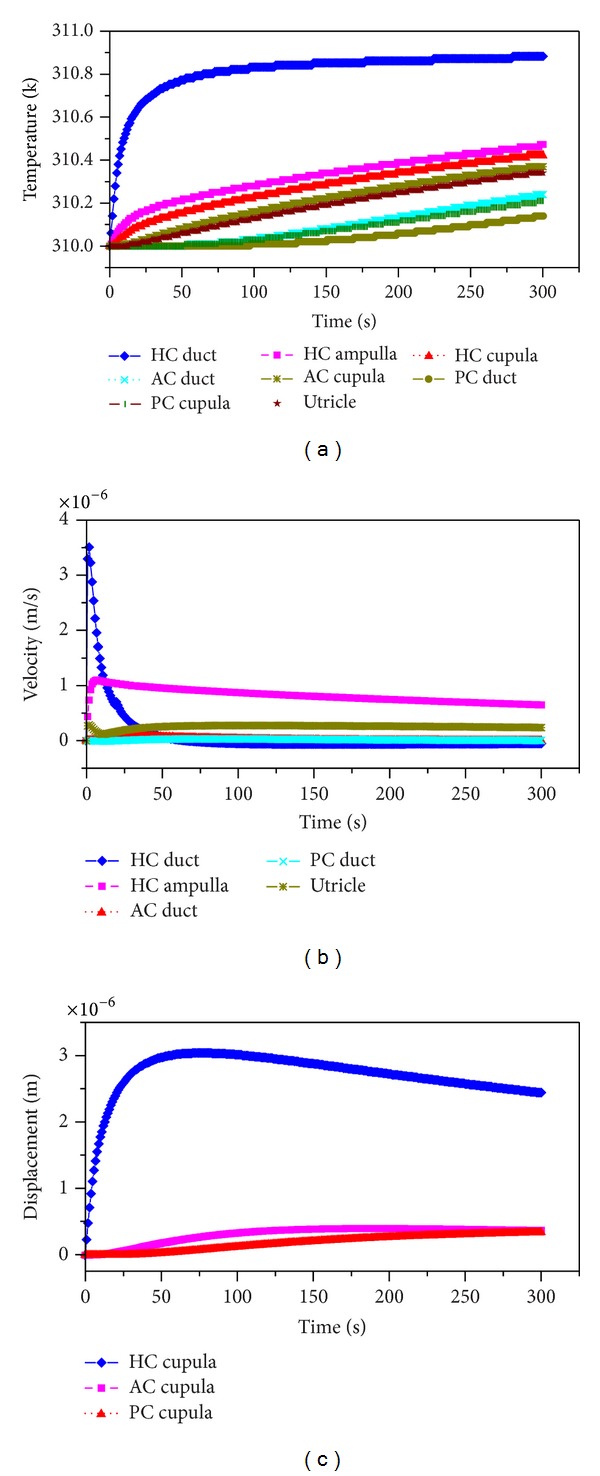
Time histories of the temperature (a), endolymph velocity (b), and cupula displacement (c) for a hot supine caloric test with Δ*T* = 1°C. The tested point in the HC duct is P1, in the HC ampulla is P2, on the HC cupula is P3, in the utricle is P4, in the AC duct is P5, on the AC cupula is P6, in the PC duct is P7, and on the PC cupula is P8. P1~P8 are defined in Figure 1(b). The velocity is positive for an ampullopetal flow in the HC duct and in the HC ampulla, for an ampullopetal flow in the AC and PC ducts, and for an utriculofugal flow in the utricle. Meanwhile, the deflection to the utricle side is positive for the HC cupula and to the canal side for the AC and PC cupulae.

Figures 2 and 3 show the dynamical response of the inner ear to the hot/supine caloric test. One second after loading, the temperature at the region near the loading area rises rapidly. This large temperature change causes a relatively large variation in the density of the endolymph. An ampullopetal flow due to the buoyancy-driven natural convection is generated in the HC duct, an ampullofugal flow is generated in the two vertical semicircular canals, and an utriculopetal flow is generated in the utricle side regions of the three cupulae. Moreover, the endolymph velocity in the HC duct peaked 6.29 *μ*m/s is far greater than the velocities in the other regions. Driven by the endolymph flow, the HC cupula deflects to the utricle side by 0.247 *μ*m, and the AC and PC cupulae undergo expansive deformations, which are approximately two orders of magnitude lower than the HC cupula deformation. Subsequently, the endolymph flows in the HC duct and in the utricle decrease while the rate of the temperature change in the HC duct decreases, after which the flow in the utricle begins to increase with the temperature rise at approximately 10 s, as illustrated in Figure 3.

At *t* = 20 s, the temperature in the vicinity of the AC cupula begins to increase. The ampullopetal endolymph flow in the HC duct weakens, while the flows in the HC ampulla and the utricle region surpass the flow in the HC duct at this time, with a maximum velocity of 4.3 *μ*m/s. The endolymph velocities in the AC and PC ducts are still far less than that in the HC duct, and the endolymph flow in the AC duct is reversed. The HC cupula continues deflecting toward the utricle side by 2.49 *μ*m, and the AC cupula deflects toward the canal side by 0.0376 *μ*m, while the PC cupula undergoes compressive deformation. At *t* = 40 s, the heat conduction continues toward the utricle and subsequently to the AC and PC ducts. The endolymph flow pattern and cupular deflection are similar to that at *t* = 20 s except for the deformation of the PC cupula, which wholly deflects to the canal side.

The temperature increased everywhere throughout the entire test, as shown in Figure 3(a), and the temperature was higher and faster with increasing proximity to the heating section. The deflection of the HC cupula to the utricle side reaches maximum at 3 *μ*m at about *t* = 70 s; this moment is defined as the peak response time. After this, the HC cupula slowly deflects back to the resting position because the temperature rise in the HC ampulla exceeds that in the HC duct, and the endolymph flow begins to move away from the ampulla. The temperatures in the AC and the PC are far lower than the temperatures in the other regions at the beginning, but heat conduction provides a considerable temperature increase. Nevertheless, both the endolymph flows in the AC and PC ducts and their cupular deformation are still relatively small. At *t* = 300 s, heat conduction has reached the entire semicircular canals system. The HC cupula slowly deforms to the utricle side by 2.44 *μ*m, and the AC and PC cupulae deform to the canal side by 0.372 *μ*m and 0.341 *μ*m, respectively. 

**Figure 4:**
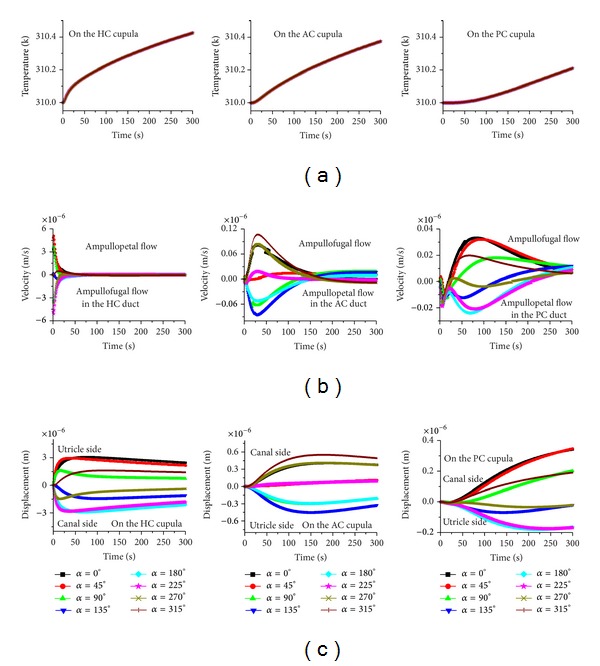
The effects of head position on the caloric responses when the gravity is parallel to the plane *α*. (a) Temperature, (b) endolymph velocity, and (c) cupula displacement.

The behavior of the system indicates that the rate of the temperature change plays a primary role in the vestibular caloric response through the buoyancy force affecting the endolymphatic natural convection. As a result, the direction of the gravitational body force, that is, the head position, can strongly affect the caloric response. Based on this reasoning, different head positions are considered in this study. When the HC plane is almost parallel to the gravity and the heat conduction is constant, the endolymph flow and the cupular deformation responses in the three semicircular canals change remarkably with respect to the head position, as shown in Figure 4. Particularly, the caloric responses in the HC are dependent upon the head position. The endolymph velocity and the cupular deformation reversed to the same extent when the head position undergoes a 180° turn. For example, the caloric responses (endolymph flow and cupular deformation) in the HC performed for a hot caloric test in the supine position (*α* = 0°) and in the prone position (*α* = 180°) are equivalent but in opposing directions. A similar phenomenon is also observed in both of the vertical semicircular canals. The deformation of the HC cupula as well as the endolymph flow in the HC duct reaches a maximum if the caloric test is performed in the supine position or in the prone position. The maximum deformation is approximately 3 *μ*m. The peak response time of the HC cupula is dependent on the head position. 

The AC and PC cupulae exhibit a hysteresis effect. The latent time is approximately 20 s for the AC cupula if the caloric test is performed in the HC plane, but it is about 40 s for the PC cupula. The change of the time constant for both cupulae is not obvious. The maximum deformation for the AC cupula is about 0.55 *μ*m, and it is about 0.34 *μ*m for the PC cupula.

**Figure 5:**
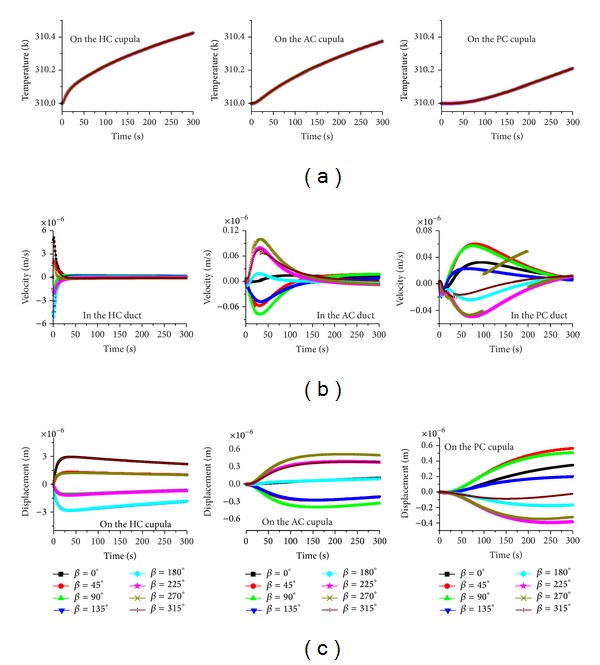
The effects of head position on the caloric responses when the gravity is parallel to the plane *β*. (a) Temperature, (b) endolymph velocity, and (c) cupula displacement.

**Figure 6:**
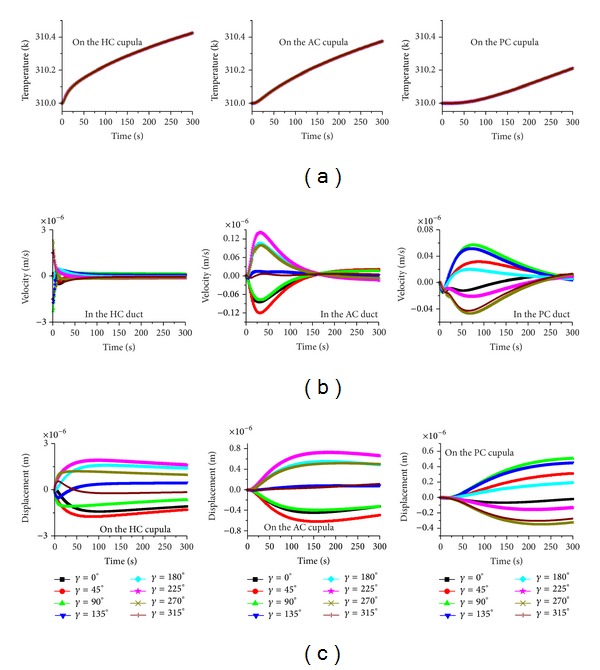
The effects of head position on the caloric responses when the gravity is parallel to the plane *γ*. (a) Temperature, (b) endolymph velocity, and (c) cupula displacement.

In order to estimate whether the caloric response in the AC of the PC can increase, plane tests for the AC (plane *γ*, as shown in Figure 1) and the PC (plane *β*) are conducted. In these two plane tests, all the patterns are similar to the HC plane test (see Figures 5 and 6). However, the maximum deformations of the HC, AC, and PC cupulae are 1.9 *μ*m, 0.72 *μ*m, and 0.5 *μ*m, respectively, for the AC plane test and 3 *μ*m, 0.51 *μ*m, and 0.56 *μ*m, respectively, for the PC plane test. There is no substantial increase in the caloric responses for the AC and PC cupulae in the AC and PC plane tests.

## 4. Discussion 

The patterns of the endolymph flow and HC cupular deflection in the supine and prone caloric tests predicted in this study are consistent with those predicted by Kassemi et al. [26] and clinical observations of the caloric nystagmus. The results in this work are therefore considered to be reliable. 

In general, the clinical caloric test is performed in the supine position with the head tilted up 30° so that the HC is placed in the vertical plane and is aligned with the gravitational field for the irrigation of warm or cold water for 40 s. In addition, the eye nystagmus is usually observed to occur after irrigating for more than 20 s. This period of time is required for the heat conduction through the human temporal bone. The latent effects of the deformation responses of both the AC and PC cupulae disclose that their contribution to the eye movements is likely to be inhibited. In other words, irrigating for 40 s is likely the optimum period to maximally stimulate the HC cupula, but the effects of the other cupulae are eliminated. This phenomenon explains why there is only a small amount of vertical and rotary nystagmus in the clinical caloric test. Therefore, the functionality of the vertical semicircular canals is not reliably evaluated in clinical tests. 

In order to effectively evaluate two vertical semicircular canals, the irrigation period must be prolonged. The optimum irrigation period is about 60 s for evaluating the AC because only the HC and the AC receive thermal stimuli during this period. More time is required for evaluating the PC. In this way, the caloric responses of the AC and the PC can possibly be distinguished, so they can be evaluated separately. However, more experiments are required to determine whether a patient can undertake this modified caloric test.

Another interesting result is that the supine or prone position tests can maximally stimulate the HC, but the caloric responses in the AC and the PC are not substantially improved by changing the tested head direction. The conventional head position is suitable for this particular caloric test. 

The main limitation of this work is that the temperature loading is exerted under ideal conditions. In fact, if the ear canal is irrigated continuously with warm water, the temperature in the segment of the HC that is closest to the bone will gradually rise, and the heat conduction will also arrive at other segments of the inner ear. As a result, this limitation affects the prediction of the endolymph flow and the cupular deformation in each canal. A more realistic model is required in the future.

## 5. Conclusion 

In this paper, an FE model is used to predict the caloric response in the three semicircular canals. The caloric test conventionally performed in clinics is proven to be the optimal test for evaluating the functionality of the HC. The results indicate that a longer period of irrigation time is required in order to stimulate the two vertical canals.

## Figures and Tables

**Table 1:** The physical and structural properties of the endolymph and cupula.

Property	Value
Cupula density (kg/m^3^)	1000
Cupula conductivity (W/m °C)	1.004
Cupula-specific heat (J/kg °C)	4186
Cupula volume expansion (1/°C)	0.00044
Cupula Young's modulus (Pa)	5
Cupula Poisson ratio	0.49
Endolymph density (kg/m^3^)	1000
Endolymph conductivity (W/m °C)	1.004
Endolymph-specific heat (J/kg °C)	4186
Endolymph dynamic viscosity (Pa·s)	0.000852
Endolymph volume expansion (1/°C)	0.00044
